# Craniotomy and Its Alternatives

**Published:** 1881-06

**Authors:** 


Craniotomy and Its Alternatives.—We are indebted to Dr. George P. Yost, of Logansville, Pa., for a copy of the April number of the *Quarterly Transactions of the Lancaster City and County Medical Society*, containing his valuable paper on "Craniotomy and Its Alternatives," read before the York County Medical Society at its recent meeting, and we could wish it were read by all those addicted to the often unnecessary operation of craniotomy. Dr. Yost has brought together an array of facts and statistics that ought to cause conviction in the mind of anyone that the unnecessary mutilation and destruction of the life of an unborn fœtus, except under the most imperious circumstances, is nothing more nor less than downright murder. It would appear that, while in England it is "resorted to once in about 200 labor cases, and in France one in 1,200," it is resorted to in Germany but "one in 1,950 cases." He says that:

"Before birth the child is just as much a living, distinct individuality as it is after. It has as perfect a right to its life as the mother has to hers. We are equally bound to save its life if we can, and we ought not only to dare, but it is our bounden duty, to put its life—that of an unborn child—into the scale against that of a being, like ourselves, accountable to the Almighty. In fact, it ought to be deeply impressed upon every practitioner that *he who destroys* the *life* of a child *without due evidence* that this is his *only* resource for saving the mother, *is a murderer.*"

This paragraph is both logical and in harmony with the dictates of humanity and common sense. We would cheerfully make several extracts from this very sensible paper, but space will allow of but one more. He states that:

"Comparing, then, the statistics of craniotomy with its elective alternatives in the actual number of *lives* saved, in the many other advantages, especially of the Porro operation, the low per cent. of mortality of ovariotomy, and the rapid strides of successful abdominal surgery, I feel that *craniotomy*, as a general operation in contracted pelvis, where we have a viable fœtus, *has had its day—a thing to be abhorred as a relic of barbarism.* Of its elective alternatives, I believe that the modified Porro method will most surely be the one to take its place, as giving the best results, and making us forget craniotomy with all its heavy attendant responsibilities."

